# Response to growth hormone in patients with *RNPC3* mutations

**DOI:** 10.15252/emmm.201809143

**Published:** 2018-06-04

**Authors:** Gabriel Á Martos‐Moreno, Lourdes Travieso‐Suárez, Jesús Pozo‐Román, María T Muñoz‐Calvo, Julie A Chowen, Mikko J Frilander, Luis A Pérez‐Jurado, Federico G Hawkins, Jesús Argente

**Affiliations:** ^1^ Department of Endocrinology Hospital Infantil Universitario Niño Jesús Madrid Spain; ^2^ Instituto de Investigación La Princesa Madrid Spain; ^3^ Department of Pediatrics Universidad Autónoma de Madrid Madrid Spain; ^4^ Instituto de Salud Carlos III CIBER de Fisiopatologia de la Obesidad y Nutriciόn (CIBEROBN) Madrid Spain; ^5^ IMDEA Food Institute CEIUAM+CSIC Madrid Spain; ^6^ Institute of Biotechnology University of Helsinki Helsinki Finland; ^7^ Genetics Unit Universitat Pompeu Fabra Barcelona Spain; ^8^ Instituto de Salud Carlos III Hospital del Mar Research Institute (IMIM) and Centro de Investigación Biomédica en Red de Enfermedades Raras (CIBERER) Barcelona Spain; ^9^ SA Clinical Genetics Women's and Children's Hospital & University of Adelaide Adelaide SA Australia; ^10^ Diabetes and Bone Research Center Institute i + 12 Complutense University and Hospital 12 de Octubre Madrid Spain

**Keywords:** Development & Differentiation, Genetics, Gene Therapy & Genetic Disease, Pharmacology & Drug Discovery

## Abstract

After 6 years of Growth Hormone (GH) therapy, three patients with a defect in minor spliceosome mRNA processing leading to an incompletely understood GH deficit present with excellent auxological response and improvement in the bone mineral density and trabecular bone structure.

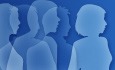

## Background

The etiology of GHD remains unknown in most cases (Alatzoglou *et al*, 2009). *RNPC3* mutations emerged as a novel cause of familial isolated GHD and pituitary hypoplasia (Argente *et al*, 2014). *RNPC3* encodes a 65‐kDa protein that is a structural component of the U11/U12 small nuclear ribonucleoprotein of the minor spliceosome (Verma *et al*, 2018). Mutations in *RNPC3* lead to structural destabilization of the 65‐kDa protein, impaired binding of U12 snRNA, and global defects in splicing of U12‐type introns (Argente *et al*, 2014; Norppa *et al*, 2018).

We describe the effects of rhGH therapy on growth, body composition, bone mineral density (BMD), and bone microarchitecture in the first three patients identified with this condition.

## Subjects and methods

Written informed consent was obtained from all subjects and their parents for all studies and their publication. Studies conformed to the principles set out in the WMA Declaration of Helsinki and the Department of Health and Human Services Belmont Report.

**Figure 1:**
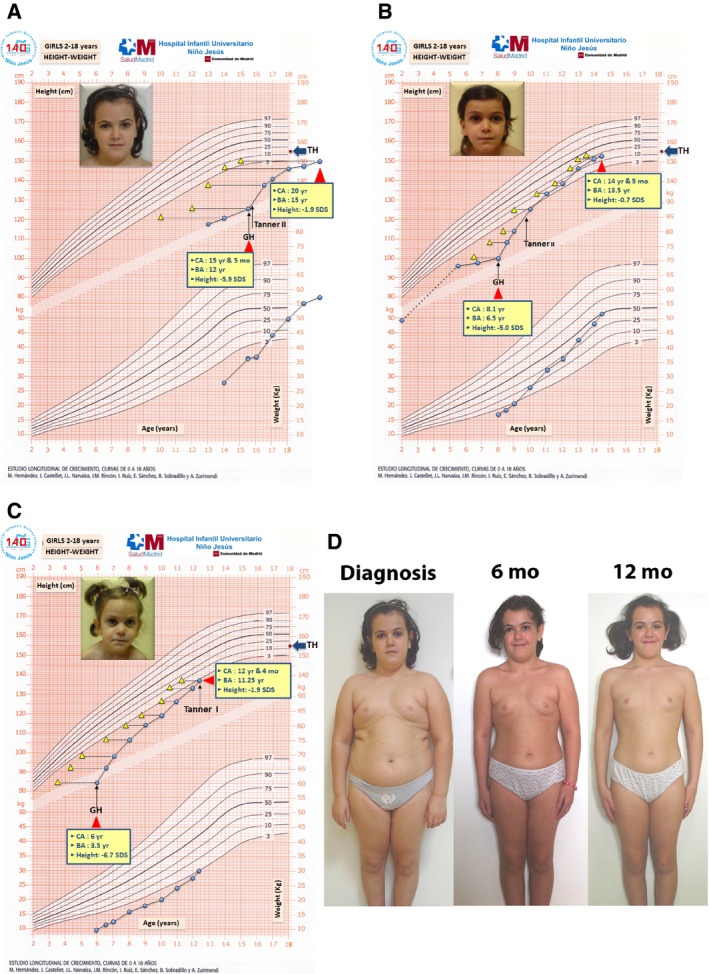
Growth charts of the three sisters after GH therapy Facial appearance at baseline and growth charts of patients 1 (A), 2 (B), and 3 (C). Blue circles represent height for chronological age, whereas yellow triangles represent height for bone age. BA, bone age; CA, chronological age; GH, start of recombinant growth hormone treatment; TH, target height. Faces of the patients reproduced with permission. (D) Changes in body fat content and distribution showing the lipolytic effect of recombinant human growth hormone treatment in patient 1 after 6 and 12 months of therapy from baseline. Reproduced with permission.

Three sisters were born to non‐consanguineous average‐height [target: 155.6 (−0.99 SDS)] Romanian parents. The father is a heterozygous carrier of a nonsense mutation (c.1504C>T, p.R502X), and the mother and only unaffected daughter are heterozygous for a missense mutation in *RNPC3* (c.1320C>A, p.P474T). The three affected girls, compound heterozygous for both mutations, were born at term with normal length and weight, developing severe postnatal growth failure, typical phenotypic features of GHD, and delayed bone age (BA; Table 1 and Fig 1). They were referred to our clinic at 15.5, 8.1, and 6.0 years of age with extremely short stature (Table 1), undetectable serum IGF‐1, IGFBP‐3 and GH after stimuli (insulin and clonidine), and no clinical or hormonal signs of associated pituitary hormone deficiencies. Anterior pituitary hypoplasia was found in MRI.

**Table 1:** Anthropometric data at baseline and after rhGH therapy onset

	Chronological age (bone age)	Height (SDS)	Growth velocity (SDS)		DXA (baseline)	DXA2	DXA3	DXA4
Patient 1								
Baseline	15.5 (12.0)	125.5 (−5.9)	—	Weight (kg)	36.7	36.8	35.9	61.3
1^st^ year	16.5 (13.0)	137.8 (−4.2)	12.8 (+31.0)	BMI (kg/m^2^)	23.3	20.75	20.91	27.1
2^nd^ year	17.5 (14.0)	143.8 (−3.1)	6.0 (+21.0)	BMD (g/cm^2^)	0.6440	0.733	0.781	1,050
3^rd^ year	18.5 (14.0)	146.4 (−2.6)	2.6 (+14.4)	BMD (Z‐score)	−3.7	−2.7	−2.2	0.4
4^th^ year	19.5	148.8 (−2.2)	2.3 (+12.5)	TBS	1,270	1,280	1,400	N.A.
Patient 2								
Baseline	8.1 (6.5)	100.4 (−5.0)	—	Weight (kg)	17.5	18.5	20.2	50.4
1^st^ year	9.0 (7.5)	114.0 (−3.2)	14.2 (+11.4)	BMI (kg/m^2^)	17.4	15.75	14.76	20.81
2^nd^ year	10.0 (9.0)	125.1 (−2.0)	11.1 (+5.5)	BMD (g/cm^2^)	0.478	0.515	0.523	0.825
3^rd^ year	11.1 (10.5)	133.2 (−1.5)	8.2 (+1.4)	BMD (Z‐score)	−1.3	−0.7	−1.0	−1.0
4^th^ year	12.0 (11.3)	138.5 (−1.6)	5.7 (−1.2)	TBS	1,260	1,270	1,360	N.A.
5^th^ year	13.0 (12.5)	146.2 (−1.2)	7.8 (+2.8)					
6 year & 6 month	14.5 (13.5)	152.9 (−0.7)	5.0 (+1.2)					
Patient 3								
Baseline	6.0 (3.5)	84.5 (−6.7)	—	Weight (kg)	9.8	11.5	12.7	36
1^st^ year	7.0 (5.0)	98.5 (−4.4)	14.6 (+9.3)	BMI (kg/m^2^)	13.7	15.77	14.76	18.90
2^nd^ year	8.0 (6.5)	106.9 (−3.6)	8.4 (+3.7)	BMD (g/cm^2^)	0.397	0.446	0.470	0.678
3^rd^ year	9.0 (7.5)	114.2 (−3.2)	7.4 (+1.1)	BMD (Z‐score)	−2.2	−1.4	−1.0	−1.1
4^th^ year	10.0 (8.8)	119.3 (−2.9)	5.4 (+0.5)	TBS	1,260	1,270	1,360	N.A.
5^th^ year	10.9 (10.0)	126.4 (−2.5)	7.1 (+1.3)					
6 year & 6 month	12.3 (11.3)	137.5 (−1.9)	7.4 (+0.5)					

SDS, standard deviation score. Bone mineral density (BMD) and trabecular bone structure (TBS) in DXA (dual‐energy absorptiometry analysis) in the three patients before (baseline) and after 6 months (DXA2), 1 year (DXA3), and 6.5 years (DXA4) of rhGH therapy onset (patient 1 received 4.5 years of treatment, and patients 2 and 3 remain on treatment). All of the DXA data refer to lumbar spine (L1–L4) (N.A., not available).

Daily subcutaneous rhGH (0.025–0.035 mg/kg/day) was prescribed, with regular clinical, laboratory, and BA (Greulich & Pyle) evaluations.

Lumbar spine BMD (LS‐BMD) and body fat percentage were measured using dual‐energy X‐ray absorptiometry (DXA Discovery Wi, software version 13.3; Hologic, Inc., Waltham, MA, USA) before and 6 months, 1, and 6.5 years after rhGH therapy onset (coefficient of variation 0.70). Data for BMD were adjusted by height‐for‐age Z‐score (Zemel *et al*, 2010).

Trabecular bone structure (TBS) was calculated from the same DXA acquisition used for LS‐BMD (TBS iNsight software, v3.0; Medimaps, France). In children, there is no international consensus of what constitutes a normal or abnormal TBS. In adults, TBS ≥ 1,350 is proposed to be normal, values between 1,200 and 1,350 are consistent with partially degraded bone, and TBS ≤ 1,200 indicates degraded bone (Silva *et al*, 2014).

## Results

### Growth, puberty, and biochemical evolution

#### Patient 1

At age 15.5 years, she was 125.5 cm (−5.9 SDS) with proportional short stature, evident central adiposity, typical facial features of GHD (Fig 1A), no signs of pubertal development (Tanner stage I), and retarded skeletal maturation (3.5 years below chronological age). On rhGH therapy, growth increased drastically, particularly during the first 2 years [growth velocity (GV) 12.8 and 6.0 cm/year, for ages 16.5 and 17.5, respectively], achieving a height of 150.3 cm at age 20 (after 4.5 years on therapy; Fig 1A).

Puberty started spontaneously shortly after starting rhGH, with menarche at age 16. However, she had oligomenorrhea, with sparse menstrual bleedings despite pubertal FSH/LH and estradiol levels and a pubertal shaped uterus with endometrium present at ultrasonography (data not shown).

#### Patient 2

At treatment onset, she was 8.1 years and 100.4 cm (−5.0 SDS), with no signs of pubertal development (BA: 6.5 years) and phenotypic features of GHD (Fig 1B). She responded intensely to rhGH, especially during the first 2 years (+14.2 and +11.1 cm/year, respectively; Table 1). At her last visit (age 14.5 years), she was 152.9 cm (−0.7 SDS), close to her target height (155.6 ± 5 cm). She started puberty (Tanner stage II) spontaneously at age 9.75 years, progressing to Tanner stage IV, but without menarche up to her last visit.

#### Patient 3

She was 6 years old (BA 3.5 years) and 84.5 cm (−6.7 SDS) at the onset of rhGH (Fig 1C). Her GV also increased dramatically during the first 2 years on treatment (+14.6 and +8.4 cm/year, respectively; Table 1). She reached the 3^rd^ centile in height at age 12.3 years, remaining prepubertal and with BA retarded 1 year, with a 4.9 height‐SDS increase after 6.5 years on treatment.

Patients 1 and 2 normalized serum IGFBP‐3 after 1 month of rhGH and IGF‐I after 6 months. In the youngest sister, IGF‐I and IGFBP‐3 did not reach reference ranges until 1 year on rhGH, remaining normal up to 4.5 years of therapy. In all patients, there was a 6‐month period during the fourth year of therapy when GV, IGF‐1, and IGFBP‐3 levels decreased (Table 1 and Fig 1) due to lack of treatment adherence.

The three siblings exhibited mild hypercholesterolemia (positive paternal family history) before therapy that did not change significantly during treatment.

### Body composition

The first year of rhGH therapy improved LS‐BMD and normalized TBS in all patients (Table 1). BMD Z‐score remained unchanged after the 1‐year DXA in patients 2 and 3, but fully normalized in patient 1. An intense lipolytic effect of rhGH treatment was observed in patient 1 during her first year on therapy, decreasing body fat from 44.1% (+2.9 SDS) to 27.2% (+0.1 SDS) (Fig 1).

## Discussion

In all three patients with GHD due to mutations in *RNPC3,* rhGH treatment was highly effective despite the severity of their short stature and considering that therapy was started after age 15 in the eldest. The improvement in height SDS after 4.5 (for the eldest) to 6.5 years on rhGH was between 4.0 and 4.9 SDS, with the two younger siblings continuing to grow. This change in height SDS is higher than the average response to rhGH in patients with isolated GHD (Darendelier *et al*, 2011 and Argente *et al*, 2014), but similar when compared with severe isolated GHD (Ranke & Lindberg, 2010 and Argente *et al*, 2014). This effect was maximum in patient 3, probably because rhGH was started at a younger age and her baseline height was more severely compromised (Ranke & Lindberg, 2010). However, the eldest sister increased her height in 24.8 cm despite her advanced chronological (15.5) and bone age (12 years) at therapy onset, with growth progressing even after menarche, achieving a 21.4‐cm pubertal growth spurt. However, this late onset of treatment might have compromised her adult height (−0.9 SDS below target height), which is below that expected for her siblings with their height centile close (patient 3) or above (patient 2) their target and still growing on therapy.

The improvements in BMD and TBS during the first year on therapy indicate that the GH‐induced rise in IGF‐I is fundamental for improving bone development, as we recently reported in patients with PAPP‐A2 deficiency (Hawkins‐Carranza *et al*, 2018). Follow‐up of the two younger sisters is required to determine whether BMD and TBS completely normalize and to investigate an eventual relationship between *RNPC3* mutations and possible impairment of the GnRH axis as suggested by the pubertal and menstrual evolution in patients 1 and 2.

The extremely positive response to exogenous GH treatment suggests that the required receptors and downstream signaling molecules are intact. Indeed, these patients showed almost undetectable GH levels after standard stimuli and basal IGF‐I, IGFBP‐3, and ALS levels suggesting that the lack of pituitary GH secretion is the underlying cause for their growth failure. Moreover, their lack of antibody production in response to this treatment further indicates an intact GH1 gene. The data and the pituitary hypoplasia observed in these patients highly suggest that the minor spliceosome plays a crucial role in the processing of genes required for somatotroph development and GH synthesis.

The positive family history of hypercholesterolemia and lack of improvement during rhGH replacement (even when the lipolytic effect of rhGH was highly evident) suggest that this finding is most likely independent from GHD.

In summary, despite the fact that the underlying mechanism by which the *RNPC3* mutations result in GHD is not completely understood, rhGH dramatically increased growth in three girls with severe isolated GH deficiency due to a defective minor spliceosome mRNA processing, determining a significant improvement in BMD, microarchitecture of the bone, and body composition.

## Conflict of interest

The authors declare that they have no conflict of interest.

## For more information

Publicly available 1,000 genomes (http://www.1000genomes.org) and 6,503 samples from exome variant server (http://www.gs.washington.edu/evs); and U12 database (U12DB, http://genome.crg.es/datasets/u12).
